# New Opportunities to Meet the Grand Challenges in Infectious Diseases

**DOI:** 10.3389/fgeed.2020.00001

**Published:** 2020-01-31

**Authors:** Chen Liang

**Affiliations:** Department of Medicine, McGill AIDS Centre, Lady Davis Institute, Jewish General Hospital, McGill University, Montreal, QC, Canada

**Keywords:** infectious disease, pathogen, genome editing, CRISPR, health

Historically, infectious diseases have taken a heavy toll on the human population. The past has repeatedly warned us that one fatal pathogen can kill millions of people. The Black Death pandemic in Eurasia took as many as 100 million lives in the fourteenth century (Cohn, 2008), and the 1918 Spanish flu killed more than 50 million individuals in less than 2 years (Taubenberger and Morens, 2019). This situation began to change in the twentieth century with the advent of two remarkable successes, antibiotics and vaccines, which have saved hundreds of millions of lives from otherwise deadly infections. It is unimaginable how many lives would have been lost if we have not had vaccines for smallpox, yellow fever, polio, and other lethal pathogens. It is incomprehensible what would happen in surgical wards if we do not have antibiotics.

One pleasant coincidence is that the tools and technologies leading to these great successes are often provided by microbes themselves: antibiotics are produced by bacteria and fungi, vaccines are often attenuated or inactivated microbes. Equally fascinating is that microbes, including viruses and bacteria, have taught us the molecular language to comprehend the most fundamental processes of life, and have inspired us to develop powerful biotechnologies to prevent and treat various life-threatening infections. One pillar of modern health science is DNA biology and recombinant DNA techniques. It is the bacteria and viruses which have taught us DNA is the genetic material and how gene expression from DNA is executed and regulated. More gratefully, we have also acquired from these microbes the molecular tools to decode DNA sequences and engineer DNA clones. Nowadays, next generation sequencing and metadata analysis have revolutionized the ways we manage infectious diseases at the levels of diagnosis, prevention, and treatment.

Despite these ground-breaking achievements, infectious diseases still lay a grave burden on public health, causing 10 to 15 million deaths annually. Attesting to this heavy global impact, six out of the 10 threats to global health, announced by WHO (World Health Organization) in 2019, are related to infectious diseases (https://www.who.int/emergencies/ten-threats-to-global-health-in-2019). These six threats include the global influenza pandemic, antimicrobial resistance, Ebola and other high-threat pathogens, vaccine hesitancy, Dengue, and HIV (human immunodeficiency virus).

It is by no accident that these infectious pathogens and related issues are listed atop the global health challenges. Influenza epidemics have been frequently recorded in human history. We are simply unable to eradicate influenza viruses from the human population partially due to their sporadic transmission into humans from their natural reservoirs of birds and other animals (Olsen et al., 2006). It has already been a challenge to produce an effective seasonal flu vaccine, and it will be a far more difficult task, if not currently impossible, to forecast and prepare for an unpredictable and yet forthcoming flu pandemic.

We have benefited from the use of antibiotics for decades. However, overuse of antibiotics and other ill medical practices have accelerated the emergence of resistance bacteria. Without a sustainable pipeline of new antibiotics, and without other effective treatments of bacterial infections, we may succumb to infections caused by multidrug-resistant pathogenic bacteria, otherwise known as superbugs. In the United States alone, 35,000 people die of antibiotic-resistant bacterial infections annually, as reported by the Centers for Disease Control and Prevention. Recent Ebola outbreaks in Central and Western Africa claimed thousands of lives, with a staggering mortality rate up to 90% (Malvy et al., 2019). If Ebola virus is not contained and instead spreads out to other regions and continents, the lives of millions of people would be at stake. Additionally, about 100 million people are infected by Dengue viruses every year in the tropical and subtropical regions, who may develop dengue fever and even die (Guo et al., 2017). HIV has killed over 35 million people since the early 1980s, and leaving another 38 million infected (sources by United Nations Programme on HIV/AIDS). In addition to these haunting infectious microbes, malaria, tuberculosis, as well as Zika virus and other emerging pathogens pose no less threat to global health.

The pressure of infectious diseases on public health has intensified over the recent years partly due to fluctuating climate change. One example is the spread of tropical pathogens to gradually warming northern territories. Dengue virus and Zika virus are carried by mosquitoes that are migrating north (Ryan et al., 2019). In history, climate change was partly blamed for the deadly Black Death pandemic (Cohn, 2008). Another key factor contributing to the quick spread of infectious pathogens is the escalating globalization which accelerates the growth of our economy, yet at the same time, exacerbates the global transmission of pathogens due to increased travels.

Whereas, non-communicable diseases such as cancers and cardiovascular illness may impact more dearly on individual health, infectious diseases and the quick transmission of microbes pose greater challenges to social life, community stability, and even national security. The outbreaks of polio in the 1950s heartbreakingly deserted playgrounds, and SARS (severe acute respiratory syndrome) in the early 2000s dreadfully interrupted international travel due to efforts to quarantine. Paralysis of our social life and structure is not inconceivable in the event of a flu pandemic if we are underprepared.

Science and technology always bring hope. As we have learned in the past, solutions are often found through interacting with our rivals. A revolutionarily powerful genetic tool has now been developed as a result of studying bacterial resistance to phage infection. This new genome editing technology, called CRISPR (clustered regularly interspaced short palindromic repeats) (Jansen et al., 2002), makes it feasible now to more efficiently and more precisely edit and rewrite the genomes of pathogens and hosts (Gasiunas et al., 2012; Jinek et al., 2012; Cong et al., 2013; Mali et al., 2013). This technological breakthrough narrows the gap between our advanced ability of reading a genome and our relatively lagging ability of editing it. We are optimistic that, with this innovative genome editing tool, radical advances will be made in response to grand health challenges posed by infectious diseases.

Prior to the discovery of CRISPR, efforts have already been made to construct genome editing tools, which have led to the innovation of TALENs (transcription activator-like effector nucleases) and ZFNs (zinc-finger nucleases) that are based on protein recognition of DNA nucleotide sequence (Gaj et al., 2013). Yet, programming these molecular machineries to target specific DNA sequence has been time-consuming due to its sophistication. In contrast, the ease of programming CRISPR effectors by altering guide RNA sequence has led to an explosion of CRISPR applications across many health science disciplines, including the field of infectious diseases.

A number of exciting successes have already been reported in diagnosing and treating infectious diseases with the application of CRISPR. One success is the rapid and ultra-sensitive detection of pathogen RNA or DNA using the CRISPR effectors. Such diagnostic platforms, including SHERLOCK (specific high-sensitivity enzymatic reporter unlocking) (Gootenberg et al., 2017) and DETECTR (DNA endonuclease-targeted CRISPR *trans* reporter) (Chen et al., 2018), are portable, use isothermal amplifications, and can be multiplexed to report co-infections. These are ideal tools for field epidemiology study.

Another successful story is the use of CRISPR to treat chronic viral infections. From time to time, our immune system fails to clear viral infections. Chronic infections of hepatitis B virus, hepatitis C virus, human papillomavirus, HIV, herpes simplex virus and others can, over time, lead to tissue damage, cancer, and ultimately death. CRISPR offers one strategy to clear chronic viral infections through targeting and removal of the viral genome. Indeed, a number of studies have already demonstrated the efficacy of CRISPR in cultured cells and in small animal models in eliminating chronic viruses (Li et al., 2018; Panfil et al., 2018; Dash et al., 2019; Jubair et al., 2019).

Will CRISPR be one solution to the quickly rising antimicrobial resistance? One immediate application of CRISPR is to target and eliminate the antibiotic resistance genetic elements, thus restoring antibiotic susceptibility (Yosef et al., 2015). CRISPR has also been programmed to knock out the bacterial virulence genes or directly eliminate the pathogenic microbes (Bikard et al., 2014). It is also conceivable that CRISPR can be exploited to boost phage therapy to treat otherwise difficult bacterial infections. One strategy can be the selection or generation of phages that are able to breach CRISPR-mediated bacterial defense, thus killing pathogenic bacteria more efficiently.

In addition to the above applications, CRISPR also provides a powerful research tool to investigate and understand infectious diseases at many levels. For example, CRISPR conveniently allows the knockout or modification of viral genes, especially in large DNA viruses, so we can better decipher their functions. Precise CRISPR-mediated alteration of host genes has become a routine practice in research labs, and this greatly accelerates the study of host factors that either facilitate or restrict viral infections. At the organism level, CRISPR-engineered animal models can be quickly generated to study pathogenesis of various infectious diseases.

The genome editing field is quickly evolving. CRISPR has upgraded from simply being a pair of DNA scissors to functionally edit single nucleotides and rewrite short DNA sequences (Kim et al., 2017; Anzalone et al., 2019). The CRISPR toolbox not only has versatile DNA editors, but also burgeoning RNA editors (Abudayyeh et al., 2017). It appears that more effective and adaptable CRISPR tools will continue to be discovered and created. Without a doubt, the field of infectious diseases, as with other health science disciplines, will continue to capitalize on this exciting and powerful molecular technology. The Infectious Diseases section of journal Frontiers in Genome Editing is dedicated to publishing innovative studies that harness CRISPR technology to tackle the most pressing challenges posed by infectious diseases.

## Author Contributions

The author confirms being the sole contributor of this work and has approved it for publication.

### Conflict of Interest

The author declares that the research was conducted in the absence of any commercial or financial relationships that could be construed as a potential conflict of interest.
